# Emergence, Dissemination and Antimicrobial Resistance of the Main Poultry-Associated *Salmonella* Serovars in Brazil

**DOI:** 10.3390/vetsci9080405

**Published:** 2022-08-03

**Authors:** Diéssy Kipper, Andréa Karoline Mascitti, Silvia De Carli, Andressa Matos Carneiro, André Felipe Streck, André Salvador Kazantzi Fonseca, Nilo Ikuta, Vagner Ricardo Lunge

**Affiliations:** 1 Institute of Biotechnology, University of Caxias do Sul (UCS), Caxias do Sul 95070-560, Rio Grande do Sul, Brazil; dkipper1@ucs.br (D.K.); andreakaroline88@hotmail.com (A.K.M.); amcarneiro@ucs.br (A.M.C.); afstreck@ucs.br (A.F.S.); 2 Molecular Diagnostics Laboratory, Lutheran University of Brazil (ULBRA), Canoas 92425-350, Rio Grande do Sul, Brazil; silvia.decarli93@gmail.com; 3 Simbios Biotecnologia, Cachoeirinha 94940-030, Rio Grande do Sul, Brazil; fonseca@simbios.com.br (A.S.K.F.); ikuta@simbios.com.br (N.I.)

**Keywords:** *Salmonella*, Brazil, Gallinarum, Enteritidis, Minnesota, Typhimurium, Heidelberg, antimicrobial resistance, poultry

## Abstract

**Simple Summary:**

Salmonellosis is a human and animal disease caused by *Salmonella*, a bacterial genus classified into different species, subspecies, and serological variants (serovars) according to adaptation to one or more different hosts (animals and humans), pathogenicity profiles, and antigenic properties. Some specific *Salmonella* serovars can spread more easily in the enteric microbiota of avian species, often causing disease in birds and/or being transmitted to humans through food (such as chicken and eggs). Antimicrobial resistance (AMR) has also been reported in poultry-associated *Salmonella* isolates due to the widespread use of antimicrobials on farms. The availability of comprehensive data on the emergence and spread of *Salmonella* serovars, as well as their AMR profiles in farms and food products in Brazil (a major producer of poultry in the World), is necessary to understand their relevance in all avian production chains and also occurrence in poultry-derived foods. This article aims to provide an overview of the genus *Salmonella* and the main serovars that emerged in Brazilian poultry over time (Gallinarum, Typhimurium, Enteritidis, Heidelberg, and Minnesota), reviewing the scientific literature and suggesting more effective prevention and control for the future.

**Abstract:**

*Salmonella* infects poultry, and it is also a human foodborne pathogen. This bacterial genus is classified into several serovars/lineages, some of them showing high antimicrobial resistance (AMR). The ease of *Salmonella* transmission in farms, slaughterhouses, and eggs industries has made controlling it a real challenge in the poultry-production chains. This review describes the emergence, dissemination, and AMR of the main *Salmonella* serovars and lineages detected in Brazilian poultry. It is reported that few serovars emerged and have been more widely disseminated in breeders, broilers, and layers in the last 70 years. *Salmonella* Gallinarum was the first to spread on the farms, remaining as a concerning poultry pathogen. *Salmonella* Typhimurium and Enteritidis were also largely detected in poultry and foods (eggs, chicken, turkey), being associated with several human foodborne outbreaks. *Salmonella* Heidelberg and Minnesota have been more widely spread in recent years, resulting in frequent chicken/turkey meat contamination. A few more serovars (Infantis, Newport, Hadar, Senftenberg, Schwarzengrund, and Mbandaka, among others) were also detected, but less frequently and usually in specific poultry-production regions. AMR has been identified in most isolates, highlighting multi-drug resistance in specific poultry lineages from the serovars Typhimurium, Heidelberg, and Minnesota. Epidemiological studies are necessary to trace and control this pathogen in Brazilian commercial poultry production chains.

## 1. Introduction

Meat consumption has been shifting towards poultry. It has been mainly driven by chicken (*Gallus gallus*) and turkey (*Meleagris gallopavo*), due to the relatively low cost of production [1]. Moreover, eggs are considered foods of high nutritional value for humans and they are widely consumed too [2]. 

Brazil is an important producer and exporter of poultry meat in the World, with volumes of 13.8 million and 4.2 million tons, respectively, in 2020. In addition, Brazil produced 172.3 thousand tons of turkey meat and 53.5 trillion eggs in this same year. Almost all stages of the poultry production chain (meat and eggs) are carried out inside the country, including farming the breeding birds (grandparent stock, hatchery, breeders), broilers, and layers. Furthermore, poultry foods are also largely processed in slaughterhouses and eggs industries in different Brazilian regions [3]. 

Foodborne bacteria have been detected in all poultry-producing regions in the World in the last seventy years [4]. Different bacteria species are the main pathogens of meat and eggs [5]. Poultry meat contamination occurs frequently by *Salmonella* spp., *Campylobacter jejuni, Campylobacter coli* and *Clostridium perfringens* [4]. Other frequent poultry foodborne bacteria include *Staphylococcus aureus*, *Listeria monocytogenes*, and *Escherichia coli* [4,5]. The bacterial genera *Pseudomonas*, *Hafnia*, *Serratia*, *Rahnella*, *Yersinia*, and *Buttiauxella* have also been detected in poultry products [6,7]. Among all these pathogenic bacteria, *Salmonella* has been the most concerning to public health. Poultry foods most commonly associated with *Salmonella* outbreaks are eggs, chicken, and other meals mixed with poultry products [4]. In addition, some *Salmonella* serovars are also associated with specific poultry diseases with huge economic losses [8]. 

Prevention and control measures are adopted in producing farms and food processing industries to prevent the occurrence of the main *Salmonella* serovars worldwide. In Brazil, *Salmonella* is also routinely controlled on farms, with vaccination and constant laboratory diagnosis to monitor the infection of the flocks and prevent transmission to poultry-derived food [9]. Despite these measures, several poultry diseases and foodborne *Salmonella* outbreaks were reported in Brazil in recent decades. This review describes the main features of the *Salmonella* genus, including serovar classification and antimicrobial resistance (AMR), as well as the emergence and spread of serovars most frequently associated with poultry in Brazil.

## 2. Classification into Serovars

*Salmonella* belongs to the Enterobacteriaceae family. It is a Gram-negative bacillus, non-spore-forming, facultative anaerobic, and generally mobile due to the peritrichous flagella. Moreover, the genus is classified into the bacterial species *S. enterica* and *S. bongori*, with the first being divided into six subspecies: *enterica*, *salamae*, *arizonae*, *diarizonae*, *houtenae,* and *indica*. *Salmonella* isolates from all these species and subspecies are also classified according to antigenic characteristics and more than 2.650 serovars were already reported [10]. Differentiation into serovars is performed by the laboratory analysis of the O (membrane lipopolysaccharides), H (flagellar proteins), and Vi (capsular polysaccharide) bacterial antigens within the White–Kauffman–Le Minor scheme. All these antigens are expressed in a specific formula for each serovar, for example “1,4,[5],12:i:1,2” (Typhimurium) and “1,9,12:g,m“ (Enteritidis) [11]. 

**Figure 1:**
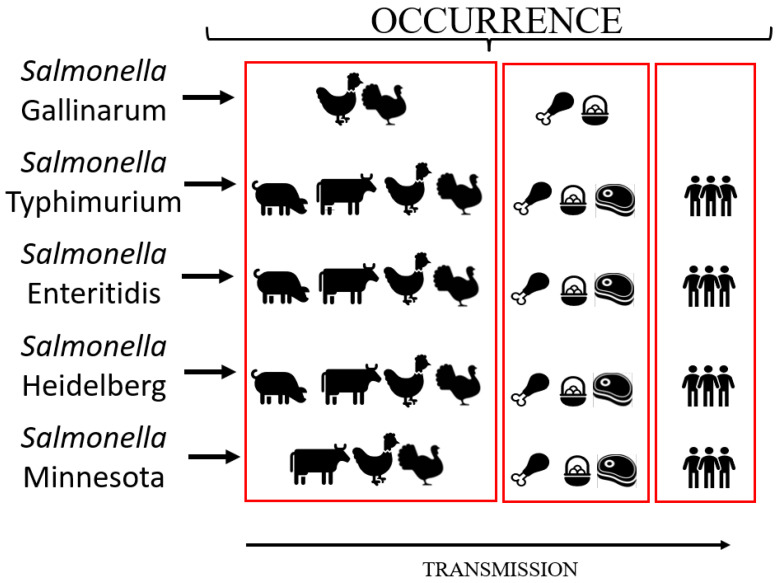
Main sources of contamination and transmission of the *Salmonella* serovars frequently detected in the Brazilian poultry production chain.

*Salmonella* has also been classified according to two specific human clinical manifestations into typhoid and non-typhoid types. The first group is composed of the etiologic agents of enteric fever, and currently includes serovars Typhi and Paratyphi, while the second group is composed of all other serovars [12]. Similarly, salmonellosis in poultry is also divided into two main groups according to the pathogenesis and avian clinical manifestations: (i) typhoid, including generalized infection by *Salmonella*, resulting in fowl typhoid (FT) and pullorum disease (PD), both caused by the serovar Gallinarum biovars, Gallinarum and Pullorum, respectively, which are highly adapted and restrict transmission among chickens (*Gallus gallus*) and a few other bird species; (ii) paratyphoid, including all *Salmonella* associated with enteric infection in birds presenting or not presenting clinical disease under special circumstances (laying period, very young or old birds, viral co-infections). This last type of salmonellosis is caused by any serovar other than Gallinarum (such as Typhimurium, Enteritidis, Heidelberg, Minnesota, etc.), which also can be transmitted to humans by direct contact (on farms, slaughterhouses, etc.) or consumption of contaminated poultry foods [13] (Figure 1).

*Salmonella* intra-serovar lineages laboratory identification is necessary for outbreak epidemiological investigations. The first method used to identify *Salmonella* isolates in outbreaks was phage typing in the 1980s [14]. Each *Salmonella* isolate from a given serovar was sorted into a unique phage type according to its reactivity against a set of specific viruses [15,16]. This procedure was more frequently used in the epidemiological surveillance of the concerning serovars Typhimurium and Enteritidis [15,16,17]. *S.* Typhimurium more disseminated definitive (phage) types (DTs) included DT49, DT104, DT135, and DT193 [18,19], while *S.* Enteritidis epidemiologically important phage types (PTs) were PT4, PT8, PT13a, and PT13 [20,21]. 

More recently, different molecular DNA-based methods have also been included in the arsenal of laboratory methods for epidemiological investigations, such as Pulsed-Field Gel Electrophoresis (PFGE), Multi-locus Sequence Typing (MLST), core genome Multi-locus Sequence Typing (cgMLST), Clustered Regularly Interspaced Short Palindromic Repeats (CRISPR), Multiple-Locus Variable-Number Tandem-Repeats Analysis (MLVA) and sequencing analysis of the Intergenic Spacer Regions (ISRs) of ribosomal RNA operons [22,23,24,25,26]. There are even some molecular techniques to identify the serovar from whole-genome sequences (WGS) data, such as *Salmonella* In Silico Typing Resource (SISTR) [24] and SeqSero [27]. *Salmonella* WGS data have also been increasingly used to evaluate single nucleotide polymorphisms (SNPs) in the complete genomes and to track specific lineages of many serovars in different stages of the poultry production chain [28,29].

PFGE is a genotyping method based on previous total DNA digestion of a specific bacterial isolate with few restriction enzymes followed by pulsed-field electrophoresis. It was largely used from the 1980s to the 2000s to track specific *Salmonella* lineages [30]. MLST allows the characterization of *Salmonella* isolates by sequencing seven housekeeping genes: *aroC*, *dnaN*, *hemD*, *hisD*, *purE*, *sucA*, and *thrA* [31,32]. It was refined and many studies are reporting the use of cgMLST, allowing the determination of the *Salmonella* sequence type based on all core genome genes [25]. According to these last two methods, *Salmonella* isolates have been currently classified into hundreds to thousands of different sequence types (STs) as an additional identification to serovar assignment [25,32]. As the number of *Salmonella* genomic sequences has increased, several STs were already reported and are now available in current specific databases. In addition, there are proposals to change the conventional nomenclature of *Salmonella* serovars, now using the genomic characteristics of the bacterial isolates and the specific ST groups [32,33].

## 3. Antimicrobial Resistance

*Salmonella* is also a matter of concern due to the occurrence of AMR in some specific serovars [34,35]. The excessive use of antimicrobials in animal/agricultural production and treatment of human/animal diseases has allowed the selection of many *Salmonella* strains resistant to one or more antimicrobials [36]. 

Antimicrobials have been administered in animal production with three main objectives: (1) to enhance animal performance, using low doses continuously throughout the feed; (2) to prevent the occurrence of pathogenic bacteria, using intermediate doses before or during critical transitions in the production process; and (3) to treat infectious animal diseases in the producing flocks/herds, usually with higher doses [37,38]. Aminoglycosides and other antimicrobial classes have been included in poultry production over time [39,40]. Tetracyclines and sulfonamides were banned as additives in animal feed in 1988, but their use for therapeutic purposes is still allowed, and are currently used to treat sick animals [41]. Data collected from 103 countries globally indicated that the Americas, Asia, and Eastern Oceania used 86% of 93,092 tons of antimicrobial agents for animals in 2017, with tetracycline and penicillin ranking at the top of the most used ones [42]. 

The prolonged use of antimicrobials has possibly increased *Salmonella* resistance to the most used classes in the poultry production chains [39,43,44]. Overall, 6 out of the 10 most frequently poultry-associated *Salmonella* serovars in the United States (Enteritidis, Montevideo, Schwarzengrund, Infantis, Thompson, and Mbandaka) have been demonstrated to be generally pan-susceptible or with resistance to few antimicrobials, whereas four (Heidelberg, Typhimurium, Kentucky, and Senftenberg) are more commonly reported as resistant to many of them [45]. Some *Salmonella* isolates from these last four serovars have also been reported as multidrug-resistant (MDR), which means, resistant to three or more antimicrobials classes [34,44,45,46,47]. 

Due to the intensive farming and the long history of antibiotic use, Brazil has reported the occurrence of AMR in different *Salmonella* serovars [48]. A study evaluating 930 WGS of different *Salmonella* serovars retrieved from the public database of the National Center for Biotechnology Information (NCBI) and published in the last four decades, demonstrated the prediction of the MDR phenotype in 58% (540/930) of the isolates, highlighting ciprofloxacin and nalidixic acid with the highest frequency rates [39]. Other recent reports have also demonstrated that MDR is frequent in *Salmonella* serovars Heidelberg and Minnesota isolated from broilers, layers, and poultry-derived food in Brazil [44,49,50,51,52].

## 4. Emergence and Dissemination 

The intestines of birds are colonized by several microorganisms that make up the host’s microbiota in a state of equilibrium. *Lactobacillus*, *Bifidobacterium*, *Streptococcus*, *Bacteroides*, *Fusobacterium,* and *Eubacterium* are frequent bacterial genera of beneficial microbiota. Imbalance of the intestinal microbiota can occur and favor the colonization by pathogenic micro-organisms, including the genera *Salmonella*, *Clostridium*, *Escherichia*, *Campylobacter*, *Staphylococcus,* and *Listeria* [53,54].

Several factors affect colonization by *Salmonella* in poultry flocks on commercial farms, including host age, genetic susceptibility, stress due to overcrowding or secondary disease, level of exposure to pathogens, intestinal microbiota competitors, and bacterial genetic factors [55,56]. Furthermore, *Salmonella* needs first to multiply in the enteric tract of some poultry in the flock, after contaminating the environment with the excretion of high bacterial loads in the litter [57]. In the host infection, *Salmonella* has to compete with other micro-organisms for a niche that provides nutrients for replication and fights against the host’s immune defenses [58]. To successfully reach the host’s gut and start the infection, the bacteria need first survive in hostile environments. *Salmonella* has developed “biological tools” to be a good competitor, comprising a set of virulence factors, plasmids, prophages, and even mobile genetic elements acquired in its evolutionary history [59,60].

One specific and very important adaptation step in the evolutionary process was the acquisition of the genetic cluster known as *Salmonella* pathogenic island 1 (SPI-1). It produces a type III secretion system necessary for the enterocyte invasion. In addition, several other gains and losses of genes occurred over time and a total of 24 different SPIs were already reported scattered into different serovars [61]. In addition to SPI-1, four other SPIs have been more well-studied: SPIs-2 to 4, which are necessary for the bacteria to multiply and survive within the host, and SPI-5, which regulates inflammation and the secretion of metabolites for the enteric phase of the disease [62]. The remaining pathogenicity islands are also necessary according to other specific environments and hosts [61,62].

**Figure 2:**
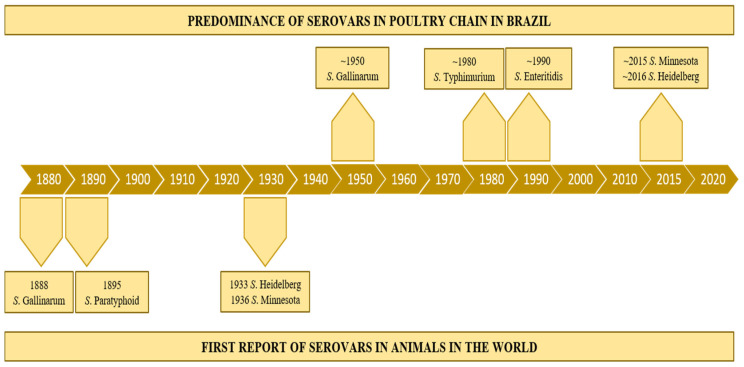
Timeline of the emergence and dissemination of the main *Salmonella* serovars in Brazilian poultry-production chain.

The emergence of a new *Salmonella* serovar in a specific ecological niche in poultry chains is frequently associated with the effective reduction in other bacteria populations (including other *Salmonella* serovars) and/or the lack of adequate immunization of the host. In the recent poultry intensive production history (over the last 100 years), “conquests and downfalls” seem to have occurred with the different serovars in the main places of poultry production in the world. In the mid-20th century, poultry diseases caused by *Salmonella* Gallinarum (including biovars Gallinarum and Pullorum) were the most concerning infections on commercial farms worldwide [63]. Some decades after, *Salmonella* Typhimurium and Enteritidis were massively detected in poultry flocks and foods (chicken, turkey, eggs), and were considered the most concerning serovars for public health [56,64,65]. Until the mid-1980s, *S.* Typhimurium was also one of the main serovars detected in animal production farms, and consequently in foods [64]. In the 1990s, *S.* Enteritidis predominated among the serovars frequently detected in avian farms and outbreaks due to the consumption of poultry foods in several countries [65,66]. More recently, other *Salmonella* serovars (such as Heidelberg, Kentucky, Montevideo, and Minnesota) were increasingly detected in specific poultry production chains and foods worldwide [67,68,69]. In Brazil, the most concerning serovars in poultry production along the time in the 20th century were the same of other poultry-producing western countries: Gallinarum, Typhimurium, and Enteritidis. Although two other serovars have been a matter of special concern in Brazilian broilers farms in this century: Heidelberg and Minnesota (Figure 2).

### 4.1. Salmonella Gallinarum

*Salmonellaenterica* serovar Gallinarum (*S.* Gallinarum) is highly adapted to avian species, causing systemic diseases in birds from poultry farms worldwide [63]. It is considered a non-motile serovar due to the absence of peritrichous flagella in all bacterial isolates. Therefore, *S.* Gallinarum presents only O-surface antigens (antigenic formula 1,9,12:-:-). This serovar is further divided into Pullorum and Gallinarum biovars, which have specific genetic, metabolic, and physiological characteristics, in addition to being responsible for two well-characterized clinical diseases in chickens (*Gallus gallus*). Recent studies have also demonstrated that *S.* Galllinarum strains probably evolved from the same ancestor of serovar Enteritidis (antigenic formula 1,9,12:g:m). The main genomic alterations include deletions and mutations, including ones related to the absence of flagellin gene expression [70,71]. 

*S.* Gallinarum bv. Pullorum causes PD, a systemic infection in young birds, frequently related to transovarial/vertical transmission. Birds with PD present bacillary white diarrhea that can progress to septicemia and death. The macroscopic lesions can include hepatitis and splenitis with white necrotic foci and purulent airsacculitis. This disease presents a more clinically concerning outcome in two- to three-week-old chickens due to the high mortality rate. The surviving birds can become asymptomatic carriers and transmit the bacteria to other chickens [72]. *S.* Gallinarum bv. Gallinarum causes FT, an acute septicemic or chronic disease that occurs most often in adult birds through horizontal transmission. Acute septicemic and/or chronic FT is responsible for 40% of death in Brazilian poultry flocks [73,74]. FT also induces significant economic losses by reducing fertility and egg production [8].

The first *S.* Gallinarum isolate was probably from the biovar Pullorum and its emergence is estimated to have occurred around 914 DC, after dispersing worldwide [75]. The initial PD outbreaks were reported in the 19th century, while the bacterium was firstly characterized in the 1900s (Figure 2). It is noteworthy that the specific evolution of this biovar resulted in the emergence of novel virulent strains with a unique ability to induce arthritis in chickens, expanding its pathogenic profile [76]. Aiming to establish sanitary measures and to achieve the control/elimination of *S.* Gallinarum bv. Pullorum, official programs in the Western countries, highlighting the United States (with the NPIP, National Poultry Improvement Plan), were implemented at the beginning of the 20th century [77]. However, this biovar became endemic in the most important poultry-producing countries worldwide and it was associated with significant economic losses in poultry farms in the 20th century [78]. PD outbreaks were reported in North America, as well as in other continents [72,79,80]. In Brazil, PD was first diagnosed in 1928 with several official notifications over time, highlighting 15 outbreaks from 2009 to 2014 [81]. 

*S.* Gallinarum bv. Gallinarum appears to have a more recent origin, but definitive evolutionary analyses have not yet been performed. FT was rarely diagnosed in the 20th century in the United States due to the NPIP implemented to control *S.* Gallinarum bv. Pullorum. The last reported FT outbreak was in 1981, so this biovar is considered eradicated in the US [82]. It has also been rare to detect FT in Europe, with only two reports in Denmark and Germany due to breeder importation [83]. However, outbreaks have been frequently reported in Asia and South America, mainly in backyard birds [84]. In Brazil, reports have described the occurrence of FT in commercial poultry farms for a long time [22,84]. The first introduction is estimated to have occurred in the mid-19th century, followed by another in the mid-20th century [85]. This biovar also seems to be highly dispersed in Brazilian poultry farms and a total of 94 outbreaks were officially reported from 2005 to 2012 (51 in 2005, 5 in 2006, 24 in 2007, 2 in 2008, 1 in 2009, and 14 in 2012) [81]. Data about outbreaks occurring after 2012 are scarcer, but FT has been frequently reported by poultry farmers [86]. In addition to the horizontal transmission of field strains that are already hosted in poultry farms, industrial incubation of contaminated eggs has also been hypothesized as a key factor for the spreading of this biovar in the whole country [87]. 

Biosecurity programs, including the elimination of *S.* Gallinarum positive poultry flocks and massive vaccination, have been the main effective tools used to eradicate FT and PD. The official Brazilian poultry health control plan (PNSA, *Programa Nacional de Sanidade Avícola*) includes rules to safeguard flocks for this serovar. Poultry flocks with suspicion of FT or PD must be screened for *S.* Gallinarum by traditional methods (bacteriology culture, biochemical tests, serology), as well as molecular biology technologies, such as polymerase chain reaction (PCR). Positive flocks have to be immediately slaughtered, and the environment must be disinfected [9]. 

*S.* Gallinarum strains also present several different genomic profiles, including lineages with several prophages, plasmids, and gene clusters coding for different AMR mechanisms [75,88]. It has also been reported that *S.* Gallinarum has accumulated many pseudogenes and virulence genes associated with specificity to chicken hosts [89]. MLST analysis has demonstrated the occurrence of at least four STs of *S.* Gallinarum bv. Gallinarum (ST78, ST331, ST470, ST762) and two of *S.* Gallinarum bv. Pullorum (ST92 and ST747) [32] (Table 1).

AMR has not been frequently reported in *S.* Gallinarum. However, the resistance to nalidixic acid, gentamicin, ciprofloxacin, kanamycin, streptomycin, enrofloxacin, and ampicillin has already been reported in different countries [90,91,92]. In addition, genes coding for AMR, as well as specific mutations in the *gyrA* gene (associated with fluoroquinolone resistance) have been observed in the bacterial genomes of *S.* Gallinarum isolates [90,91]. In Brazil, *S.* Gallinarum appears to present AMR to nalidixic acid, ciprofloxacin, enrofloxacin, and tetracycline, but not for beta-lactams [93] (Table 1). *S.* Gallinarum bv. Gallinarum isolates have additionally demonstrated resistance to azithromycin and quinolone/fluoroquinolone [93,94]. Higher resistance in the more recent *Salmonella* isolates than in samples obtained in the past was also observed and associated with the increased use of antimicrobials in the poultry farms [93]. Noteworthy, the association of some resistant *S.* Gallinarum isolates with the historical indiscriminate use of antibiotics has already been demonstrated [75,92]. Antimicrobial therapy is also described to treat PD and FT, especially in small-scale commercial layer flocks [95].

### 4.2. Salmonella Typhimurium

*Salmonellaenterica* serovar Typhimurium (*S.* Typhimurium) infects a wide range of hosts and it is the most frequent serovar isolated from intensive-producing animals and foods worldwide. In addition to having several lineages, this serovar (antigenic formulae 1,4,[5],12:i:1,2) has also monophasic and aphasic flagellar variants with slightly different antigenic properties (1,4,[5],12:i:-/1,4,[5],12:-:1,2/1,4,[5],12:-:-), but very similar genetic and metabolically activities. These few antigenic differences have resulted in several problems in epidemiological investigations, making tracking these variants a real nightmare [96]. Importantly, *S.* Typhimurium is frequently motile due to its peritrichous flagella, which include the flagellin proteins FliC (phase 1 antigen) and FljB (phase 2 antigen) [97]. Most isolates are biphasic, expressing both flagellins in different physiological conditions. However, monophasic variants of phase 1 or phase 2 are frequently detected, since lacking the capacity of flagellar antigen production is a bacterial strategy to evade the immune system of the host animals [96]. Furthermore, this serovar also has a very long evolutionary history associated with human and animal infections, resulting in a high diversity of pathogenicity, virulence, AMR profiles, and host adaptation [98].

*S.* Typhimurium was the first non-typhoid concerning serovar isolated in humans infected by contaminated foods. In the mid-1950s, it was isolated from duck eggs consumed by patients presenting severe enteric infection in Europe [99]. It was also the most frequent serovar associated with animal and human outbreaks in the United Kingdom from 1941 to 1970, with a steady increase to a maximum of 85% of salmonellosis cases in 1954 [100]. Additionally, it was detected in cattle, poultry, swine, and sheep samples (Figure 1). In livestock, this serovar was the most frequent in turkeys and also presented in cattle [101,102]. It spread to other livestock before its global dissemination in the 1990s [102]. *S*. Typhimurium and its variants have continued to disseminate in animals and in humans being the top concerning serovar in most regions of the World [98,103,104,105].

Previous epidemiological investigations traced this serovar with phage typing and *S*. Typhimurium DT104 was one of the most disseminated worldwide. This type was associated with cattle hosts, but also detected in swine, birds, and wild animals [106]. In the 1990s, there was a global epidemic of DT104 in humans, being the most common DT in Europe, Asia, and America [102,106]. Human outbreaks by this phage type were caused by direct animal transmission, imported food, travel abroad, and environmental reservoirs [107]. Recent WGS analyses have provided more evidence that it probably emerged from an antimicrobial susceptible ancestor in ∼1948 and became MDR in ∼1972 through horizontal transfer of the 13 kb *Salmonella* genomic island 1 (SGI-1) [108]. Despite the epidemiological importance of *S.* Typhimurium DT104 for public health, other DTs (for example, DT193 and U288) have also been largely disseminated in livestock, foods, and, consequently, they infected humans too [109,110]. In addition, monophasic *S.* Typhimurium 1,4,[5],12:i:- has also been a matter of concern worldwide, including in Brazil [111,112]. In addition to being responsible for human salmonellosis outbreaks in America and Europe, it was also isolated from different animals and foods [113,114,115,116].

In poultry production, *S.* Typhimurium and its variants have already been detected in layers and broilers [117]. The contamination by this serovar can occur at multiple steps along the food chain, including production, processing, distribution, retail marketing, handling, and final preparation [118]. In Brazil, it is largely reported that *S.* Typhimurium was a very common isolate in non-human sources (including broilers, layers, and poultry-derived foods) before the 1990s [119]. With the wide spread of *S.* Enteritidis at the end of the 1980s and the beginning of the 1990s, *S.* Typhimurium isolations declined in non-human sources in Brazil for some years [64,120] (Figure 2). However, it was still classified as the most common serovar isolated from humans [121]. *S.* Typhimurium monophasic variants (mainly 1,4,5,12:i:-) were also frequently detected in human, animal, and food samples in this same period [111,112,116]. More recent studies have demonstrated the detection of this serovar in livestock and animal-derived foods, including poultry meat and eggs [122,123]. 

The implementation of biosecurity procedures in farms and industries to prevent food contamination has contributed to the reduction in the incidence of salmonellosis [56,124,125]. Additionally, the use of vaccines for *S.* Typhimurium in poultry flocks and swine herds has helped to prevent the dissemination of this pathogen [126,127,128].

*S.* Typhimurium genetic, antigenic, and metabolic diversity has been evidenced by the large amount of WGS data generated in recent years. Several lineages were characterized, some of them highly adapted to specific hosts. Now it is well-known that *S.* Typhimurium is a complex group of slightly different sequence types, such as ST19, ST313, ST213, ST128, and many others [32,49,129]. Some of the main lineages seem to be more adapted to specific hosts [98] (Table 1). 

AMR has been reported in several *S.* Typhimurium isolates. Since the 1990s, MDR *S.* Typhimurium isolates have been frequently observed [102]. AMR seems to have benefitted *S.* Typhimurium DT104 to spread more quickly [108]. MDR isolates have also been associated with a higher risk of invasive infection, longer illness, increased frequency and duration of hospitalization, and a higher risk of death than antimicrobial susceptible strains [130,131]. In Brazil, MDR *S.* Typhimurium frequency has also increased over time [19]. Furthermore, MDR *S.* Typhimurium-specific lineages of swine-origin have been reported in farms and foods [132,133]. Most *S.* Typhimurium isolates obtained from poultry and chickens show resistance to several antimicrobials, including colistin [49,134,135,136]. The resistance genes detected in these isolates are presented in Table 1.

### 4.3. Salmonella Enteritidis

*Salmonellaenterica* serovar Enteritidis (*S.* Enteritidis) is highly adapted to avian species without causing severe clinical signs in most poultry-producing flocks. However, this non-typhoid serovar can occur in high concentrations in chicken meat and eggs, and can cause human foodborne outbreaks [137]. *S.* Enteritidis presents a specific surface structure with the expression of the two flagellar phases (antigenic formulae 1,9,12:g:m), so isolates are generally motile. Monophasic and even aphasic flagellar variants can be also rarely detected (1,9,12:g:-/1,9,12:-:m/1,9,12:-:-). *Salmonella* surveillance data showed that the occurrence of *S.* Enteritidis in foods increased worldwide from the 1970s to the 1980s [138]. Furthermore, epidemiological investigations have demonstrated that this global increase was related to the consumption of eggs and poultry meat [139].

*S.* Enteritidis emerged as the primary cause of foodborne outbreaks in the World in the mid-1980s [65]. The increased frequency of salmonellosis by this serovar was associated with the consumption of avian-source foods, such as eggs and undercooked chicken meat [56,138,139,140]. It was so hypothesized that *S.* Enteritidis filled the ecological niche vacated by the eradication of *S.* Gallinarum (biovars Pullorum and Gallinarum) from domestic fowl in many poultry-producing countries. Importantly, these two serovars share a common immuno-dominant somatic antigen. Enteric colonization, as well as flock immunity generated by the infection with the two biovars of *S.* Gallinarum, probably prevented an earlier emergence of *S.* Enteritidis in poultry flocks worldwide [65,141].

*S.* Enteritidis was initially traced by phage-typing and the different outbreaks were caused by PT4, PT8, and PT13a. In the United States, PT8 and PT13a were the most common in the northeast, south, and mid-west, while PT4 was predominant in the western states [142]. In Europe, PT4 was the most frequent one in *S.* Enteritidis isolated from chicken carcasses [140,143]. Therefore, most studies concluded that PT4 was more frequent in Europe, while PT8 and PT13 were prevalent in the United States [144]. In South America, the predominance of PT4 was demonstrated, followed by the less known PT7 and PT9 [145,146]. Overall, 150 *S.* Enteritidis foodborne disease outbreaks were reported only in Argentina between 1986 and 1993 [147]. In Brazil, human salmonellosis cases by this serovar were detected in all geographic regions in the late 1980s [64]. A recent study with WGS data reinforced the high increase in *S.* Enteritidis bacterial population in the second half of the 1980s and beginning of the 1990s, associated with the high frequency of this pathogen in the poultry production chain in this country [148]. 

Diagnostic tools, as well as biosecurity and control measures, were implemented to avoid the high spreading of *S.* Enteritidis. Monitoring and sanitization plans were introduced in all stages of poultry production, including breeding flocks, hatcheries, broiler flocks, and slaughter establishments [149]. In addition, layers and breeders’ flocks have been vaccinated, contributing to a significant reduction in *S.* Enteritidis contamination in the table egg industry and broiler processing plants [149,150,151,152]. In Brazil, preventive measures established by the PNSA were adopted in poultry farms, slaughterhouses, and eggs industries to reduce the prevalence of *Salmonella* spp. and to establish an adequate level of consumer protection [9]. Additionally, egg quality assurance programs were also implemented to avoid food contamination. All these procedures reduced the occurrence of *S.* Enteritidis in commercial farms and poultry-derived foods, improving public health [150,152].

*S.* Enteritidis strains present several different genomic profiles, including many lineages with some specific genetic and metabolic characteristics. MLST analysis has demonstrated the occurrence of more than 15 STs, including ST11 (the most frequent and largely disseminated), ST183, ST136, ST310, ST814, and others [32,148] (Table 1). 

This serovar has also been characterized by low resistance to antimicrobials in Brazil. However, some *S.* Enteritidis isolates from foodborne outbreaks and hospitalized patients showed an MDR profile [122,131]. *S.* Enteritidis isolates from poultry-derived products and foods showed AMR mainly to sulfonamide, trimethoprim-sulfamethoxazole, nalidixic acid, streptomycin, gentamicin, and tetracycline [153,154,155,156]. AMR genes are further detailed in Table 1. 

### 4.4. Salmonella Heidelberg

*Salmonellaenterica* serovar Heidelberg (*S.* Heidelberg) was firstly isolated from humans in the city of Heidelberg, Germany, in 1933 [157] (Figure 2). Later it was demonstrated to infect livestock, mainly poultry. It is frequently characterized as a biphasic motile serovar (antigenic formulae 1,4,[5],12:r:1,2), but monophasic and aphasic flagellar variants (1,4,[5],12:r:-/1,4,[5],12:-:1,2/1,4,[5],12:-:-) have also been reported [11]. 

Epidemiologically, *S.* Heidelberg is a serovar often detected in human foodborne salmonellosis [158,159,160]. Human infections are more frequently reported in hospitalized patients from North America than in other regions of the World [159,160]. It is estimated that more serious disease occurs in approximately 13% of human infections by this serovar [161]. However, the septicemic disease is rare, only occurring in some immunocompromised patients, the elderly, and young children [162,163]. 

Foodborne outbreaks are usually linked to the consumption of poultry-derived food [164]. *S.* Heidelberg has been frequently detected in chicken, eggs, and ground turkey [165,166,167,168,169]. However, it was also identified in other livestock and foods [170,171,172] (Figure 1). The outbreaks by this serovar have been reported worldwide [173,174,175,176,177,178,179,180]. 

In Brazil, this serovar was firstly detected in foods in 1962 [181]. It was also observed in different sources and regions of Brazil in the following four decades [181]. More recently, it has been increasingly detected in Brazilian broiler farms and, consequently, in chicken carcasses, generating economic losses for the poultry producers [182]. *S.* Heidelberg has also been very frequently isolated in poultry slaughterhouses. RASFF (Rapid Alert System for Food and Feed) reported that 50% of the *Salmonella* positive cases in chicken meat were from the serovar Heidelberg between 2013 and 2017 [183].

*S.* Heidelberg has a high ability to adapt to poultry farm environments [182,184]. Bacterial cells remain viable in the poultry litter for long periods, resisting a wide range of temperatures and pHs, as well as producing biofilms [184,185,186]. It has been a real challenge to remove this serovar from poultry farms and slaughterhouses in Brazil [182].

Recent epidemiological studies have used MLST to identify *S.* Heidelberg strains and four main STs have been reported: ST15, ST2071, ST7556, and ST3377 [29,34,44,49,177,187,188,189,190,191]. ST15 is the most frequent and it could be further divided into different lineages according to phylogenomic analyses [44]. This is also the most frequent ST detected in the Brazilian poultry production chains [29,34,44,49] (Table 1).

This serovar has also shown AMR to several antibiotics, raising concern among veterinary and public health authorities [69,191,192]. Most *S.* Heidelberg strains are resistant to tetracycline, nalidixic acid, and ampicillin, as well as some other antimicrobials [49,69,135,193,194,195,196,197,198] (Table 1). Furthermore, an MDR profile has been common in most isolates [49,135,194,195,197]. In a temporal comparative analysis, it was demonstrated that 46.1% of *S.* Heidelberg isolates were resistant to only one class of antimicrobials in 2005, while 100% of them were MDR in 2009 [199]. Brazilian *S.* Heidelberg isolates have demonstrated a high frequency of resistance (>50%) to streptomycin, nalidixic acid, tetracycline, cefotaxime, ampicillin, amoxicillin, cefoxitin, amoxicillin-clavulanate, and ceftiofur [197]. This high AMR has further been detected in raw chicken meat exported to Portugal and the Netherlands [29,34]. The most frequent AMR genes detected in *S*. Heidelberg are presented in Table 1. 

### 4.5. Salmonella Minnesota

*Salmonellaenterica* serovar Minnesota (*S.* Minnesota) is a motile serovar with the expression of the two-phase flagellar antigens (antigenic formulae 21:b:e,n,x) frequently detected in the natural environment. So, it has been detected in different sources, such as natural water samples, plants, livestock farms, and foods [200,201] (Figure 1). It can also infect humans and animals, but reports of outbreaks of this serovar are not so common [202,203].

Historical data demonstrate an association of this serovar with a few small outbreaks [202,203]. More recently, it was associated with other foodborne salmonellosis cases [204,205]. In addition to the usual gastroenteritis, typhoid-like illnesses can rarely occur [206]. 

*S.* Minnesota was first isolated in a turkey from a poultry farm located in the state of Minnesota, United States, in 1936 [207] (Figure 2). In Europe, it was first isolated from spray-dried eggs in England in 1947 [208]. It was also the most prevalent serovar in broilers from Belgium poultry farms [209]. *S.* Minnesota has raised concerns after the increase in the number of isolations in the poultry production chain in Brazil [50,69]. This serovar was the tenth most identified in avian salmonellosis from 2004 to 2008, but it became the second one in 2010 in Brazil. More recently, it was the most serovar isolated from drag swabs in broiler farms [69]. There are also additional reports of *S.* Minnesota in samples of Brazilian chicken meat [210].

The ability to produce biofilms has also been evaluated in isolates of *S.* Minnesota from poultry sources. Most isolates showed *invA*, *lpfA*, and *agfA* genes. In addition, genes linked to apoptosis induction (*avrA*), oxidative stress (*sodC*), and quorum sensing (l*uxS*) were also identified in samples of broiler slaughtering plants in Brazil, demonstrating adapting to adverse conditions. Furthermore, it has been demonstrated that S. Minnesota is a moderate-intensity biofilm producer [211].

Five main STs have been assigned to *S.* Minnesota: ST548, ST7557, ST7558, ST285, and ST3088 [34,47,49,50]. ST548 is the most frequent one, but ST3088 has also been detected in the poultry production chains [40,49,50] (Table 1). It has also been demonstrated that *S.* Minnesota strains currently circulating in Brazilian poultry can be divided into two lineages: SM-PLI and SM-PLII [50].

*S*. Minnesota strains more recently isolated have shown AMR to several antibiotics, including amoxicillin, ampicillin, cefazoline, cefoxitin, ceftazidime, ceftiofur, ceftriaxone, cephalothin, chloramphenicol, ciprofloxacin, clavulanic acid, gentamicin, nalidixic acid, neomycin, penicillin, streptomycin, sulfamethoxazole, sulfonamide, tetracycline, and trimethoprim [40,49,212,213,214,215,216] (Table 1). Furthermore, most isolates have shown an MDR profile [40,49,213,214,215]. Bacterial resistance genes have also been reported (Table 1).

**Table 1:** Phenotypic resistance, genotypic resistance, and ST already reported of the main *Salmonella* serovars from the Brazilian poultry-production chain.

Serovar and Variants	Phenotypic Resistance	Genotypic Resistance	STs	References
Gallinarum (1,9,12:-:-)	Ampicillin, azithromycin, ciprofloxacin, enrofloxacin, fluoroquinolone, gentamicin, kanamycin, nalidixic acid, streptomycin, and tetracycline.	*gyrA*, *aadA* and *aadB*	78, 92, 331, 470, 762, 747	[32,90,91,92,93,94,95]
Typhimurium (1,4,[5],12:i:1,2/ 1,4,[5],12:i:-/ 1,4,[5],12:-:1,2/ 1,4,[5],12:-:-)	Aminoglycoside, ampicillin, aztreonam, cefepime, ceftriaxone, chloramphenicol, ciprofloxacin, colistin, doxycycline, fluoroquinolone, gentamicin, nalidixic acid, streptomycin, sulfamethoxazole, sulfonamide, tetracycline, and trimethoprim.	*aac(3)-lla*, *aac(3)-lld*, *aadA1*, *aadA2*, *aph(6)-ld*, *bla_CTX-M-2_, bla_TEM-1B_*, *dfrA1, floR, mrc-1*, *strA*, *strB*, *sul1*, *sul2*, *tet(A)*, *and tet(B)*	19, 128, 213, 313	[32,49,130,136,137]
Enteritidis (1,9,12:g:-/ 1,9,12:-:m/ 1,9,12:-:-)	Gentamicin, nalidixic acid, streptomycin, sulfonamide, tetracycline, and trimethoprim-sulfamethoxazole.	*aac(3)-Iva*, *aac(6′)-Iaa*, *aph(3″)-Ib*, *aph(4)-Ia*, *aph(6)-Id*, *mdf(A)*, *tet(34)*, *tet(A)*	11, 183, 136, 310, 814	[32,149,154,155,156,157]
Heidelberg (1,4,[5],12:r:1,2/ 1,4,[5],12:r:-/ 1,4,[5],12:-:1,2/ 1,4,[5],12:-:-)	Amoxicillin, ampicillin, aztreonam, cefepime, cefotaxime, cefoxitin, ceftazidime, ceftiofur, ceftriaxone, cephalothin, chloramphenicol, ciprofloxacin, clavulanic acid, colistin, doxycycline, florfenicol, gentamicin, meropenem, nalidixic acid, pefloxacin, penicillin, quinolone, streptomycin, sulfamethoxazole, sulfonamide, tetracycline, tobramycin, and trimethoprim.	*aac(3)-Via*, *aadA1*, *aadA8*, *aph(3′)-Ia*, *bla_CMY-2_, bla_CTX-M_*, *bla_CTX-M-2_*, *bla_CTX-M-8_*, *bla_TEM-1B_*, *cmlA1*, *dfrA15*, *fosA7*, *mdf(A)*, *mphB*, q*nrB1*, *strA*, *strB*, *sul1*, *sul2*, *sul3*, *tet(34)*, *tet(A)*	15, 2071, 3377, 7556	[29,34,44,49,69,136,178,185,190,191,193,195,196,197,198,199]
Minnesota (21:b:e,n,x)	Amoxicillin, ampicillin, cefazoline, cefoxitin, ceftazidime, ceftiofur, ceftriaxone, cephalothin, chloramphenicol, ciprofloxacin, clavulanic acid, gentamicin, nalidixic acid, neomycin, penicillin, streptomycin, sulfamethoxazole, sulfonamide, tetracycline, and trimethoprim.	*aadA1*, *ant(3″)-Ia*, *aph(3′)-Ia*, *aphA1*, *bla_CMY-2_*, *bla_CTX-M_*, *bla_CTX-M-8_*, *bla_TEM_*, *mdf(A)*, *qnrB19*, *qnrB5*, *sul2*, *tet(A)*	285, 548, 3088, 7557, 7558	[34,47,49,50,213,214,215,217]

### 4.6. Other Salmonella Serovars

Several more serovars were already detected in the poultry production chains in Brazil in recent decades. Serovar Infantis appears to be the most frequent one, followed by Schwarzengrund, Senftenberg, Mbandaka, Hadar, and Newport [49,69,121,124,213,216].

*Salmonellaenterica* serovar Infantis (antigenic formulae 6,7,14:r:1,5) has been reported in humans, poultry farm environments and foods [69,124,218,219]. It is one of the 15 most isolated serovars from human sources [217,218,219]. In the Brazilian poultry chain, *S.* Infantis has been isolated at a low frequency [27,49,69,216,220]. The bacterial isolates showed AMR to different antibiotics, such as sulfonamide, tetracycline, and amoxicillin [219]. 

*Salmonellaenterica* serovar Schwarzengrund (antigenic formulae 1,4,12,27:d:1,7) was already isolated from chicken carcasses, broiler farms, frozen chicken cuts, chicken, poultry slaughterhouses, and feed factories in different Brazilian states [69,213,216,221]. The isolates presented AMR to sulfonamide, tetracycline, and amoxicillin [213], as well as a frequent MDR profile [49,216]. 

*Salmonellaenterica* serovar Senftenberg (antigenic formulae 1,3,19:g,[s],t:-) has also been detected in chicken carcasses, broiler farms, poultry environments, slaughterhouses, and food in Brazil [69,121,213,216,221,222]. Isolates were resistant to cefoxitin, ciprofloxacin, enrofloxacin, nalidixic acid, and trimethoprim-sulfamethoxazole [216,222].

*Salmonellaenterica* serovar Mbandaka (antigenic formulae 6,7,14:z_10_:e,n,z_15_) was detected in Brazilian broiler farms, poultry slaughterhouses, feed factories, and chicken carcasses [69,213,216,221]. The isolates showed resistance to sulfonamide, norfloxacin, and amoxicillin [213]. 

*Salmonellaenterica* serovar Hadar (antigenic formulae 6,8:z_10_:e,n,x) has been isolated from foodstuff, broiler chicken, poultry slaughterhouses, and chicken carcasses [213,216,223]. AMR was observed in amoxicillin, chloramphenicol, nalidixic acid, nitrofurantoin, tetracycline, streptomycin, sulfazotrim, and sulfonamide [213,223]. 

*Salmonellaenterica* serovar Newport (antigenic formulae 6,8,20:e,h:1,2) was already isolated from turkeys, broiler chicken, and poultry slaughterhouses [124,213,216]. The isolates showed AMR to different antimicrobials and MDR profile [213,216]. 

*Salmonella* from several more serovars (Abatetuba, Abony, Carrau, Grumpensis, Idikan, Isangi, Orion, Ouakam Rochdale, Saphra, etc.) have also been rarely isolated from food (chicken), food-producing animals (broiler), and environmental samples (slaughterhouse) collected in a Brazil [224]. In general, these serovars also presented AMR genes encoding resistance to quinolones, third-generation cephalosporin, tetracycline, aminoglycoside, sulfonamide, and fosfomycin, respectively [224].

## 5. Prevention and Control

In Brazil, official standards to prevent and control *Salmonella* are ruled by the National Poultry Health Program of the Ministry of Agriculture, Livestock, and Supply (MAPA, *Ministério da Agricultura, Pecuária e Abastecimento*) [9]. Feces and drag or boot swabs from the flocks are routinely collected in poultry farms and submitted to laboratory analysis with different methods (bacteriology culture, biochemical tests, serology, molecular biology assays, etc.) to detect and identify *Salmonella* serovars. In slaughterhouses and egg industries, self-control programs must be carried out to monitor the contamination by *Salmonella* spp. from the acquisition of the feedstock to the final food products [9].

Some antimicrobials have also been banned as additives and antibiotic-free (ABF) strategies have been implemented in the poultry production chains [225,226,227,228]. There are several alternatives to be used as growth promoters, such as medicinal plants, probiotics, prebiotics, and organic acids [225]. Formic acid, an extensively studied organic acid, has been reported to limit infection with *Salmonella* and other foodborne pathogens when used in the poultry diet [229]. In addition, it has also been recommended that rigorous management on the farms to avoid *Salmonella* infection, including quality control of the water consumed, biosecurity efforts, and the overall organization of the flocks [225]. 

ABF strategies can include feeding-based and non-feeding-based strategies to control *Salmonella* infection in poultry flocks. The first includes prebiotics, probiotics, synbiotics, postbiotics, and phytobiotics; while the second focuses on the use of bacteriophages, *in ovo* applications, and vaccines [228]. Prebiotics are usually administered to induce a modulating effect on the gut microbiota, increasing the growth of resident beneficial bacteria [230,231]. Probiotic bacteria (alone or in combination) have been used to control *Salmonella* infections during poultry production [232,233], improving production performance [234]. Strategies based on symbiotics may trigger some mechanisms involved in the inhibition or reduction in clinical signs caused by *Salmonella* [228]. Postbiotics involve the use of non-viable bacteria to provide benefits to poultry health [235]. Phytobiotics have also been shown to contribute to improving poultry performance, increasing nutrient uptake and carcass quality [236]. Bacteriophages have already been used against *S*. Enteritidis, *S*. Hadar, and *S*. Typhimurium, presenting interesting results [237]. Vaccination is part of the biosecurity protocol on farms to prevent the spread of diseases, such as *Salmonella* Gallinarum, Enteritidis, and Typhimurium serovars [238,239,240]. The administration of probiotics, prebiotics, and vaccines *in ovo* were shown to be effective to control *Salmonella* [228]. New technologies, including all the methods to study the omics (genomics, metagenomics, transcriptomics, proteomics, metabolomics) are useful tools for a better understanding of *Salmonella* metabolic arsenal [228].

Other studies have also compared ABF with conventional production, including animal welfare analysis [226,241,242,243]. As expected, the rate of *Salmonella* with MDR isolated from flocks of laying hens fed excluding antibiotics was significantly lower than that of chickens fed with them in conventional diets [242]. In addition, the prevalence of *Salmonella* with MDR in retail chicken meat was lower in packages categorized as “low or no antibiotic use” [243]. 

## 6. Conclusions

The dissemination of many *Salmonella* serovars has caused a huge impact on livestock production chains worldwide for decades. The five main poultry-associated serovars detected in Brazil over time (Gallinarum, Typhimurium, Enteritidis, Heidelberg, and Minnesota) demonstrated to be adapted to chicken hosts with different pathogenicity and antigenic properties. They have also presented different AMR profiles. *Salmonella* serovars presented specific dissemination waves in some restricted geographic regions or worldwide in the past, requiring plans to control the epidemics as well as to avoid economic losses on the farms and/or the occurrence of concerning foodborne outbreaks. In Brazilian poultry production, programs were also paramount for controlling the different *Salmonella* serovars epidemics. 

Furthermore, current intensive poultry production in Brazil presents a perfect scenario for the emergence of novel concerning *Salmonella* serovars. Continuous epidemiological surveillance is necessary to track all serovars and lineages, mainly those presenting AMR. It is also necessary to eliminate the use of antimicrobials in poultry production to reduce the emergence and dissemination of *Salmonella* lineages with AMR.

## Data Availability

Not applicable.
